# Impact of COL11A1 Mutations on Tumor Mutational Signatures and Immune Microenvironment in Head and Neck Squamous Cell Carcinoma

**DOI:** 10.7759/cureus.81629

**Published:** 2025-04-02

**Authors:** Austin Y Wang, Jolin X Cheng, Jing Xiao

**Affiliations:** 1 Biology, Conestoga High School, Berwyn, USA; 2 Biology, Charlotte Latin School, Charlotte, USA; 3 Biostatistics, Regeneron Pharmaceutical Inc, Basking Ridge, USA

**Keywords:** col11a1, gene mutation, head and neck squamous cell carcinoma (hnscc), tumor immune microenvironment, tumor mutational burden

## Abstract

Targeted immunotherapy can significantly improve the survival rates of head and neck squamous cell carcinoma (HNSCC) patients; however, only a minority of patients respond favorably to such treatments. In this study, we investigate the association between the tumor mutational burden (TMB) and clinical characteristics of HNSCC, as well as the impact of COL11A1 gene mutations on the immune microenvironment in head and neck cancer (HNC). In our analysis of HNSCC patient data from The Cancer Genome Atlas (TCGA) database, we found significant differences in TMB across various clinical features. Furthermore, a high TMB was associated with poorer survival outcomes. Despite its relatively high mutation frequency, the clinical significance of COL11A1 has not been fully explored. Our analysis of COL11A1 mutations in HNSCC and their functional impact found that COL11A1 mutations are associated with poor survival outcomes. Additionally, we observed a close correlation between COL11A1 mutations, reduced immune cell infiltration, and altered expression levels of chemokines. Further enrichment analysis suggested that COL11A1 mutations may alter the tumor immune microenvironment by affecting immune-related pathways, such as leukocyte activation and chemokine signaling. Finally, we evaluated the effect of the COL11A1 mutation on immune infiltration. Using a variety of immune infiltration algorithms, we found that the mutation of COL11A1 was associated with lower levels of immune cell infiltration. In summary, the present study explored the potential role of COL11A1 mutation in the progression of HNSCC, and our findings provide a potential therapeutic target for better therapeutic outcomes in HNSCC.

## Introduction

Head and neck cancer (HNC) is a collective term for cancers that may arise in various locations but primarily affect the oral cavity and larynx. It is a prevalent disease worldwide with an increasing incidence, particularly for oral cancer [1]. HNC exhibits high occurrence rates in developing and developed countries and is commonly diagnosed in older populations [2]. However, there is a trend toward younger patients due to factors such as human papillomavirus (HPV) infection and betel nut consumption [3]. The prognosis for HNC patients is generally poor, with data from The Cancer Genome Atlas (TCGA) indicating a five-year survival rate of approximately 40% [4].

Squamous cell carcinoma is the most prevalent pathological type within the realm of head and neck malignancy, with its incidence constituting a significant proportion of up to 90% [5]. For patients diagnosed early with head and neck squamous cell carcinoma (HNSCC), the standard clinical treatment typically involves a combination of surgical resection and radical radiation therapy. However, patients with advanced HNSCC encounter a high risk of local recurrence and distant metastasis. The current treatment methods are not only limited in efficacy but also associated with significant adverse reactions, leading to a generally poor prognosis. Consequently, there is a pressing need to investigate more effective therapeutic strategies and innovative therapeutic targets.

There is a strong correlation between HPV and certain types of HNCs, particularly an escalating link between HPV type 16 and such cancers [6]. Instances of HNCs attributable to HPV infections have demonstrated a marked upward progression in regions such as Northern Europe and North America [7]. Currently, the primary technical instruments for the detection of HPV16 include PCR and the confirmation of P16 protein expression via immunohistochemistry. Generally, patients diagnosed with HNCs accompanied by HPV16 infection exhibit a comparatively favorable prognosis [8]. Consequently, the status of HPV infection has been incorporated as a critical reference parameter for the clinical staging of oral cancer.

Patients with malignant tumors in the head and neck often exhibit significant genomic variations, which are considered key drivers in the induction and progression of cancer [9]. This is particularly evident in HNSCC, where the pathogenesis is closely associated with structural changes in cellular DNA [10]. Some genomic variations result in the loss of normal proliferation and apoptosis regulatory mechanisms in cells, thereby triggering abnormal cell proliferation and tumor formation. Furthermore, specific gene mutations can affect the infiltration patterns of immune cells [11]. Notably, there is a significant correlation between the TMB, the degree of immune cell infiltration, and the response to immunotherapy [12]. In particular, the loss of function in genes such as JAK1 and LKB1 has been proven to be associated with tumor immune escape mechanisms [13]. Additionally, mutations in normal stem cells or progenitor cells may lead to the formation of cancer stem cells, thereby triggering uncontrollable proliferation processes and promoting tumor formation [14].

In summary, HNSCC is a common malignant tumor. In recent years, targeted immunotherapy has brought new hope for improving the survival rate of HNSCC patients. However, only a small proportion of patients respond well to this treatment, indicating the need for a deeper understanding of the factors that affect treatment outcomes. The main objectives of this study are twofold: (1) to describe the clinical relevance and prognostic significance of tumor mutational burden (TMB) in different HNSCC subgroups and (2) to elucidate the mechanistic connection between COL11A1 mutations and immune microenvironment remodeling. The motivation for this study stems from the current lack of consensus on the use of COL1A1 as a prognostic marker for HNSCC, as well as the unexplored potential of COL11A1 mutations as immune evasion regulators. Our research provides insights that could inform the optimization of treatment strategies for HNSCC.

## Materials and methods

Public cancer database

All the public cancer databases employed in this study were procured from open-access data disseminated by TCGA, the International Cancer Genome Consortium (ICGC), and the Gene Expression Omnibus (GEO). Specifically, the data obtained from TCGA encompasses multidimensional information, including gene expression profiles, gene variant data, and patient clinical information. The raw data from TCGA was accessed through the UCSC Xena platform [15], and the Kaplan-Meier Plot function provided by this platform was employed to analyze the correlation between gene mutations in the donor-centric cohort of ICGC and patients' clinical information. Furthermore, the Kaplan-Meier Plot function was used to examine the association between gene mutations in the ICGC database and the survival rate of patients.

Joint analysis of genomic information and clinical information

The cBioPortal platform [16] (*https://www.cbioportal.org*) was utilized to comprehensively analyze mutation frequency details in head and neck tumors and expose associations between mutations and clinical parameters by integrating patient clinical data. Specifically, the platform was used to demonstrate the relationship between a patient's TMB and clinical characteristics, and the connection between TMB levels and patient survival. The association between specific mutations and the survival prognosis of the TCGA patients is further disclosed with the help of cBioPortal's survival curve plotting capabilities. The platform's Grouping Information Comparison tool was also employed to acquire and visualize information on the frequency of gene mutations in patients, including a display of specific mutation sites in the COL11A1 gene. For high-frequency mutated genes, the mutation type information is sourced from the cBioPortal website. Additionally, the categorization of patient groups and the comparison of differences in gene expression levels between groups were accomplished by using the mRNA analysis function in the "Comparison/Survival" section of cBioPortal. 

A mutation oncoplot of the top 30 mutated genes in the TCGA database for HNC patients was obtained through an online tool at *http://113.44.3.163/plot_basic_maf_oncoplot_135_en* [17], which provides visualization for understanding the distribution of these pivotal mutations.

Evaluation of tumor stemness index in TCGA-head and neck squamous carcinoma (HNSC) and assessment of HPV status

Drawing on the study by Malta et al. [18], we employed two well-established methods to evaluate the stemness characteristics of TCGA-HNSC tumors: an mRNA expression-based stemness index, predicated on the level of mRNA expression, and a DNA methylation-based stemness index. Guided by these two indices, we classified the samples into two categories: high stemness and low stemness based on the median, respectively. We then conducted a group analysis of TMB on the cBioPortal platform.

Moreover, this study incorporated previous research findings [19] to establish the HPV infection status of patients within the TCGA-HNSC dataset, whereby the patient group was divided into HPV-positive and HPV-negative groups. Subsequently, we compared the differences in the TMB between these two subgroups using the cBioPortal platform to investigate the correlation between HPV infection status and tumor genomic instability.

Enrichment analysis

Utilizing the grouping comparison function of the cBioPortal platform, we separated the patients into two groups: those with a COL11A1 gene mutation and those without. We then analyzed the difference in gene expression between these two groups. Subsequently, genes with significant expression differences between the two groups were selected from the obtained differential gene list, and enrichment analysis for molecular function, cellular component, and biological process was performed based on the Gene Ontology (GO) database. This analysis process was executed through the online tool Metascape [20], accessible at *https://metascape.org*. Regarding the gene enrichment analysis (GSEA) portion of the result set, we employed the pre-ranked GSEA function from the eVITTA platform [21], available at *https://tau.cmmt.ubc.ca/eVITTA/easyGSEA*, to identify and screen biological regulatory pathways that are significant within the dataset.

Analysis of immune infiltration

For the quantitative evaluation of the immune microenvironment in TCGA patients with HNSCC, we utilized multiple algorithms. Information pertaining to the correlation of gene mutations with immune cell infiltration and chemokine levels, acquired through the TISIDB database [22], which is maintained by the University of Hong Kong and can be accessed at *http://cis.hku.hk/TISIDB*. Based on the ESTIMATE algorithm of immune cell infiltration data from the MD Anderson Cancer Center of Bioinformatics Platform (*https://bioinformatics.mdanderson.org/estimate*). Subsequently, we categorized the patient population according to the median estimated score of each individual and conducted a comparative analysis of gene mutation frequencies between these groups using the cBioPortal platform.

In addition, to assess the immune infiltration of patients utilizing the single sample gene set enrichment analysis (ssGSEA) algorithm, we established the composition of the gene set drawing on the research paper by Luo et al. [23].

Protein interaction network

The Protein-Interacting Network Map, constructed to elucidate intricate relationships between essential genes in two core pathways, was developed utilizing the authoritative online database STRING (accessed at *https://string-db.org*). In the network, each line represents a potential protein interaction.

Single-cell sequencing results

We procured the single-cell sequencing data analysis results, published by GSE103322 and GSE150321, from the online TISCH2 dataset (accessed at *http://tisch.comp-genomics.org*) [24]. This includes a comprehensive annotation of the data and a heat map representation of gene expression.

Statistical significance

In this study, unless otherwise explicitly stated, all statistical significance tests were conducted using a p-value of less than 0.05 as the criterion for determination.

## Results

Tumor mutational burden (TMB) in HNC

**Figure 1:**
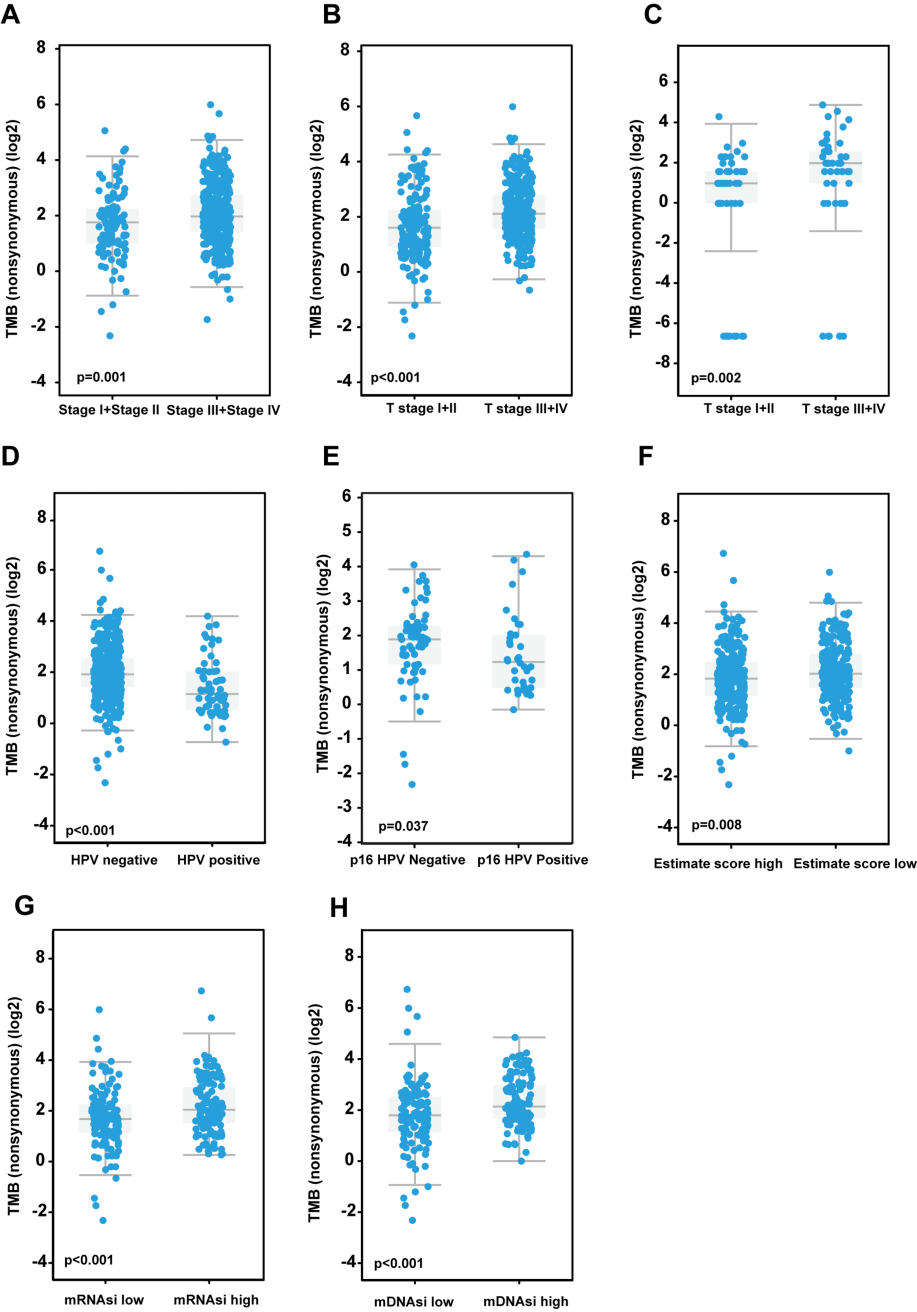
Trends in tumor mutational burden (TMB) among head and neck cancer patients TCGA: The Cancer Genome Atlas Program; HNSC: head and neck squamous carcinoma; HPV: human papillomavirus (A-B) TCGA samples validate the TMB levels across different tumor stage groups of head and neck cancer, highlighting the disparities across various T stages. (C) Data from JAMA Oncol 2016, available on cBioPortal, confirm the variations in TMB at different T stages. (D) In the TCGA cohort, the TMB levels of HPV-negative patients with HNSC were significantly higher than those of HPV-positive patients. (E) The HPV infection status in TCGA-HNSC was determined using p16 clinical methods. The box plot illustrates that the TMB in HPV-negative patients was significantly higher than in HPV-positive patients. (F) The TMB levels of HNSCC patients in the TCGA-HNSC cohort with a low estimate score were significantly higher than those with a high estimate score. (G-H) Correlation dot plots based on the mRNA expression-based stemness index (G) and the DNA methylation-based stemness index (H) demonstrate a significant positive correlation between tumor stemness and TMB. The tests of significance in this section are the Wilcoxon test

TMB constitutes a pivotal genetic characteristic identified in tumor samples, indicating genomic stability in patients. To investigate the characteristics of TMB in HNC, we analyzed the TMB distribution across various clinically significant groups of patients diagnosed with this type of cancer. First, we investigated the distribution of the TMB across various tumor staging groups. Based on the clinical data of patients from the TCGA database, patients in stages I and II were classified as the low-grade staging group, whereas those in stages III and IV were categorized as the high-grade staging group. We obtained the TMB-level data for both groups from the cBioPortal website. The results revealed significant disparities between the two groups. Specifically, the low-grade staging group demonstrated lower levels of TMB (Figure 1A), indicating more stable genomic features.

Additionally, we utilized the TCGA database to investigate the correlation between different T-stage data in the TNM classification and TMB. A higher T-stage indicates a larger tumor size and more extensive invasion of adjacent tissues. Our findings demonstrated that tumors with lower T-stages possess a lower TMB (Figure 1B). Comparable results have been noted in third-party databases (Figure 1C, data derived from the JAMA Oncology dataset published in cBioPortal in 2016). Moreover, to explore the correlation between HPV infection status and TMB, we procured the HPV status of patients from the HNSCC project in TCGA, as reported in the study by Malta et al., and analyzed the TMB levels in relation to different HPV statuses. The analysis revealed that the TMB in HPV-negative patients was significantly higher than that in HPV-positive patients (Figure 1D). Furthermore, this conclusion was supported by the p16 clinical detection method (Figure 1E). 

**Figure 2:**
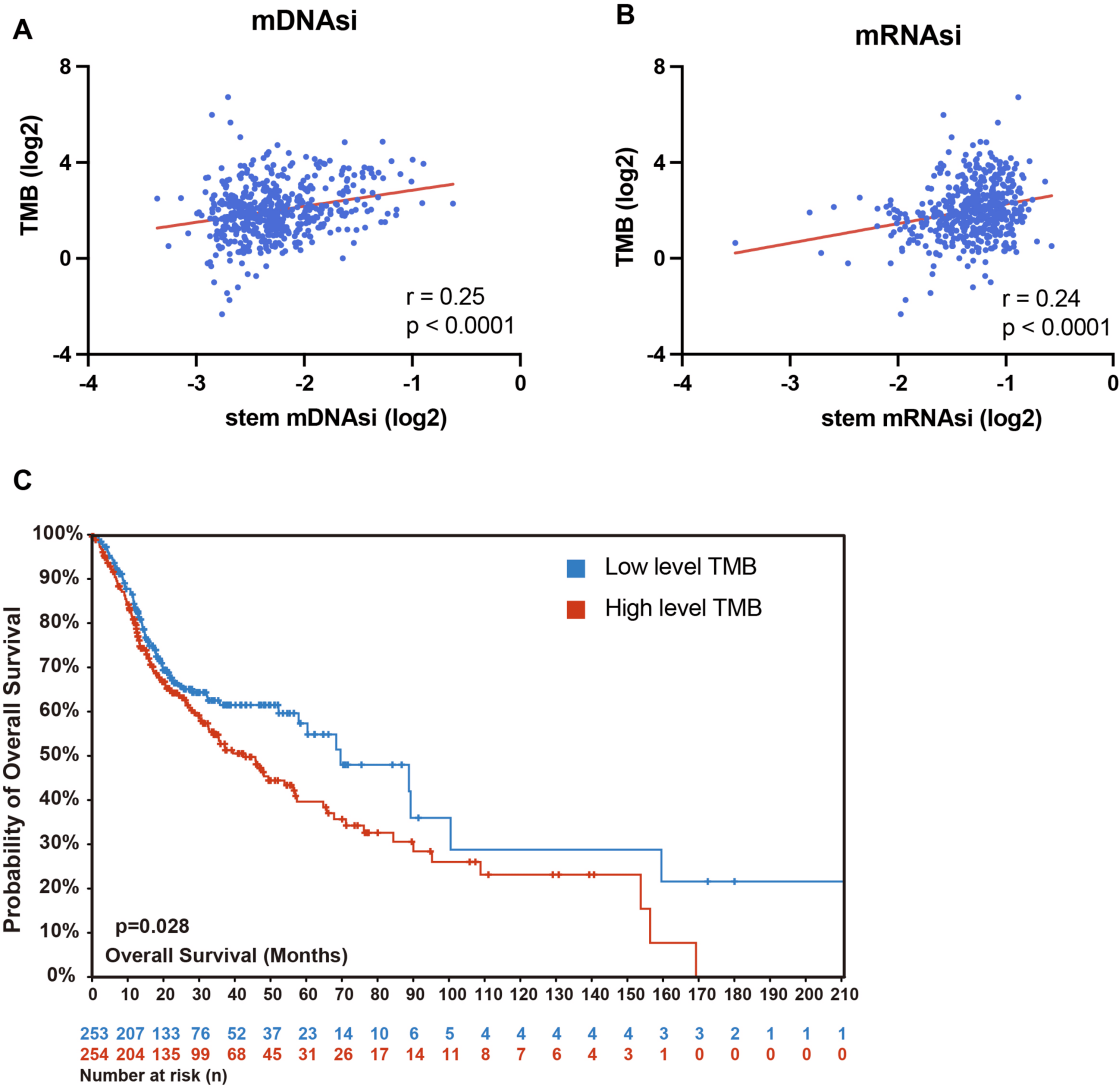
Relationship between TMB and HPV status or tumor stemness TMB: tumor mutational burden; HPV: human papillomavirus; HNSC: head and neck squamous carcinoma (A-B) Correlation dot plots based on the mRNA expression-based stemness index (Figure A) and the DNA methylation-based stemness index (Figure B) demonstrate a significant positive correlation between tumor stemness and TMB. Pearson's correlation coefficient (r) was used to evaluate the correlation between the variables. (C) The TCGA-HNSC database reveals that the survival prognosis for patients with high TMB mutation levels was significantly poorer than that of patients with low TMB mutation levels. Survival curves were compared using the log-rank test

To examine the impact of the tumor immune microenvironment and the significance of TMB as a predictor of immunotherapy efficacy, we categorized patients into two groups based on the median estimated score: a high immune cell infiltration group and a low immune cell infiltration group. The results indicated that TMB was significantly higher in the low infiltration group compared to the high infiltration group (Figure 1F). Moreover, the acquisition of stemlike properties is recognized as a key feature of tumor progression [25]. Thus, we further analyzed the relationship between stemness scores and TMB in patients with HNC. Both the RNA expression-based stemness index (Figure 1G) and the DNA methylation-based stemness index (Figure 1H) revealed that patients with higher stemness scores also exhibited higher TMB. Correlation scatter plots (Figures 2A, 2B) further confirmed a significant positive correlation between the tumor stemness index and TMB. Additionally, we classified patients into high and low mutation groups based on their TMB and generated the corresponding survival curves. The results demonstrated that patients in the high mutation group had significantly worse survival outcomes compared to those in the low mutation group (Figure 2C).

COL11A1 is a commonly mutated gene in HNC

**Figure 3:**
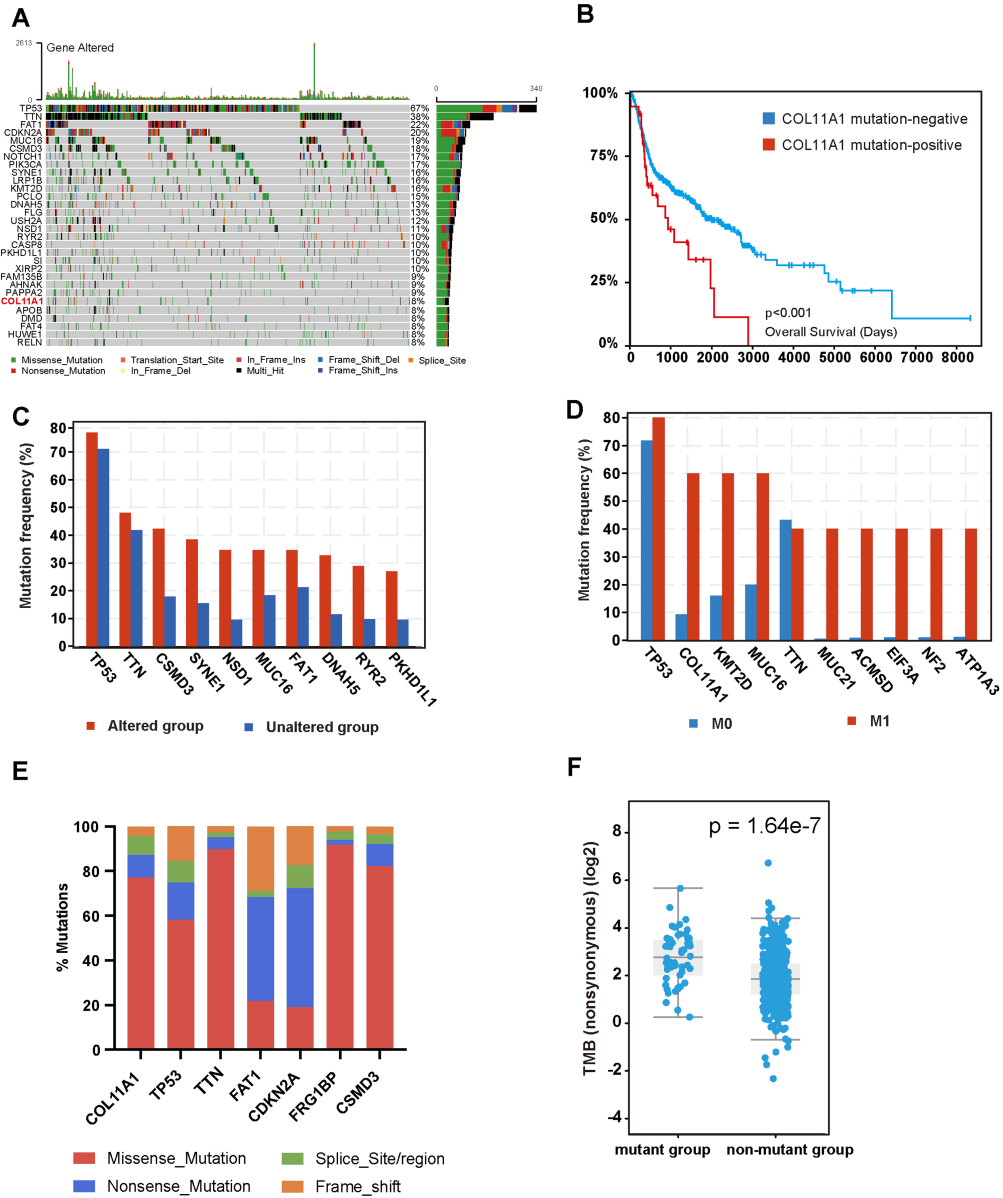
Mutation of COL11A1 in HNSC HNSC: head and neck squamous carcinoma; TCGA: The Cancer Genome Atlas; ICGC: International Cancer Genome Consortium (A) Oncoplot profiles the common genetic mutations in HNSC patients in TCGA-HNSC studies, with COL11A1 having a higher mutation frequency. Different colors represent different mutation types. (B) According to the ICGC database, the survival prognosis of patients with COL11A1 mutation was significantly lower than that of the nonmutant group, and the difference in survival curves between the two groups was tested by the log-rank test. (C) There were significant differences in the mutation frequency of hotspot mutant genes between the COL11A1 mutant group and the nonmutant group, and the data were derived from the TCGA-HNSC study. (D) Patients were divided into metastatic and nonmetastatic groups based on the M stage of TCGA-HNSC, showing significant differences in the frequency of gene mutations, including COL11A1, between the two groups. (E) In the TCGA-HNSC study, the mutation types of common mutated genes were statistically analyzed, among which the missense mutation type of COL11A1 was the most common. (F) In the TCGA-HNSC study, the TMB in the COL11A1 mutant group was significantly higher than in the nonmutant group

We used online tools (accessed at *http://113.44.3.163/plot_basic_maf_oncoplot_135_en*) to map the patients with HNSCC in TCGA (Figure 3A). It was observed that the TP53 gene exhibited the highest mutation frequency, with approximately 67% of patients demonstrating this gene mutation. Furthermore, TTN, FAT1, and other genes also displayed relatively high mutation rates, which have been extensively documented in the literature. However, despite the 8% mutation rate of the COL11A1 gene, studies pertaining to this gene are limited. Considering this, we aimed to investigate the potential correlation between COL11A1 mutations and disease progression in HNSCC. First, we initially gathered mutation and survival data from the ICGC database for patients with HNSCC. Based on the mutation status of the COL11A1 gene, patients were categorized into mutation-positive and mutation-negative groups. By conducting a survival curve analysis (Figure 3B), we found that the survival duration of patients in the COL11A1 mutation-positive group was significantly shorter compared to the mutation-negative group. This suggests that this gene mutation may be associated with a poorer prognosis.

Furthermore, our analysis demonstrated significant disparities in the frequency of gene mutations between the COL11A1-mutated and nonmutated groups. In particular, samples harboring the COL11A1 mutation exhibited a higher mutation rate in commonly mutated genes in HNC, such as TP53, TTN, and CSMD3, as depicted in Figure 3C. We also conducted a comparative analysis of gene mutation frequencies among patients with and without metastasis, which revealed that several genes, including COL11A1, exhibited higher mutation frequencies in metastatic cases (Figure 3D). To comprehend the characteristics of the variation types of these commonly mutated genes, we analyzed the proportion of mutation types of these genes. The results indicated that the mutation of the COL11A1 gene primarily manifested as a missense mutation, and its splice site or splicing region exhibited a higher mutation frequency than other genes (Figure 3E). Given the concurrent presence of COL11A1 mutation with high-frequency mutations of multiple genes, we further investigated the correlation between COL11A1 mutation status and TMB. The findings revealed that the TMB of the COL11A1 mutant group was significantly higher than that of the nonmutant group (Figure 3F).

Enrichment analysis of COL11A1 mutation-related genes

**Figure 4:**
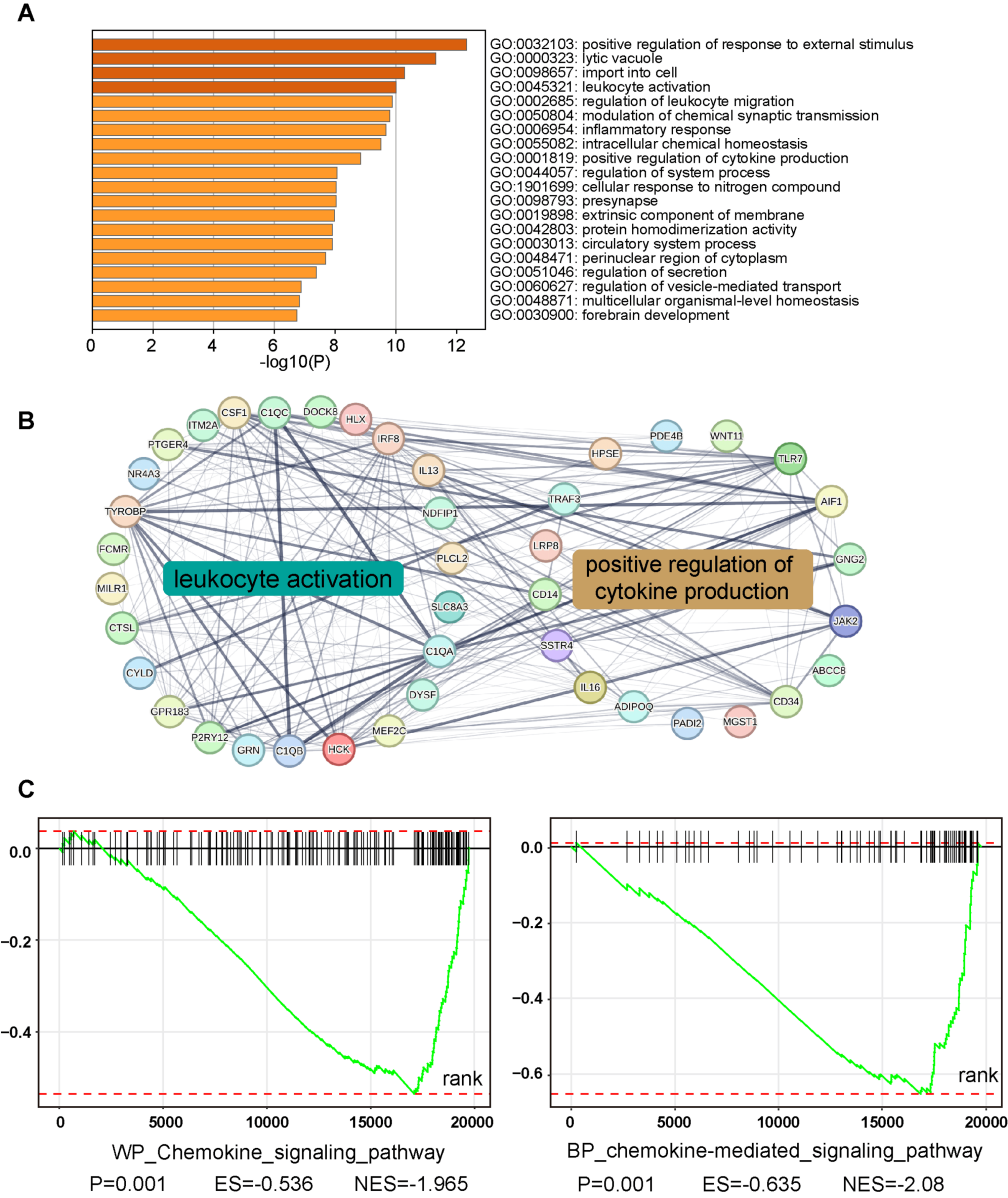
Enrichment analyses of genes associated with COL11A1 mutation (A) Depicted via a bar graph, the enrichment of distinct pathways enriched with differentially expressed genes linked to COL11A1 mutations is illustrated. (B) An interaction network visualizes the interplay among pivotal genes implicated in both leukocyte activation and positive regulation of cytokine production pathways. Each node symbolizes a gene, while the edges denote potential interactions, with line thickness reflecting the strength of confidence in such interactions. (C)The Gene Set Enrichment Analysis (GSEA) plot exhibits the enrichment of gene sets connected to COL11A1 mutations. Significant enrichments were found in the “Chemokine Signaling Pathway” from WikiPathways and the “chemokine-mediated signaling pathway” within the Gene Ontology (GO) database

The abovementioned study revealed that the COL11A1 gene exhibits a high mutation frequency in HNSCC. Despite the limited research on its specific mutation patterns, it may bear significant clinical implications. In an attempt to explore the molecular pathways potentially implicated in the effects of COL11A1 mutation, we sought to identify the disparities in gene expression between the mutant and nonmutant groups, providing the foundation for subsequent analyses. Initially, we screened for genes with statistically significant (p < 0.05) differential expression values between the COL11A1 mutant and nonmutant groups. Then, we conducted a GO enrichment analysis on these genes. The analysis unveiled that the differentially expressed genes between the two groups were enriched in numerous immune-related pathways, including leukocyte activation, regulation of leukocyte migration, and cytokine production (Figure 4A). These functional gene sets also displayed intricate and strong interaction networks (Figure 4B).

Furthermore, through GSEA on chemokine-related pathways across different datasets, we found these pathways generally exhibited high normalized enrichment scores (NES) and were significantly enriched in the context of COL11A1 mutations (Figure 4C). These studies imply that COL11A1 mutations may play a pivotal role in modulating the tumor immune microenvironment. These findings underscore the significance of further investigations into the specific mechanisms of COL11A1 mutation in the pathogenesis and progression of HNC, as well as its potential clinical applications.

Significant correlation between COLL11A1 mutations and tumor immune infiltration and chemokine expression

**Figure 5:**
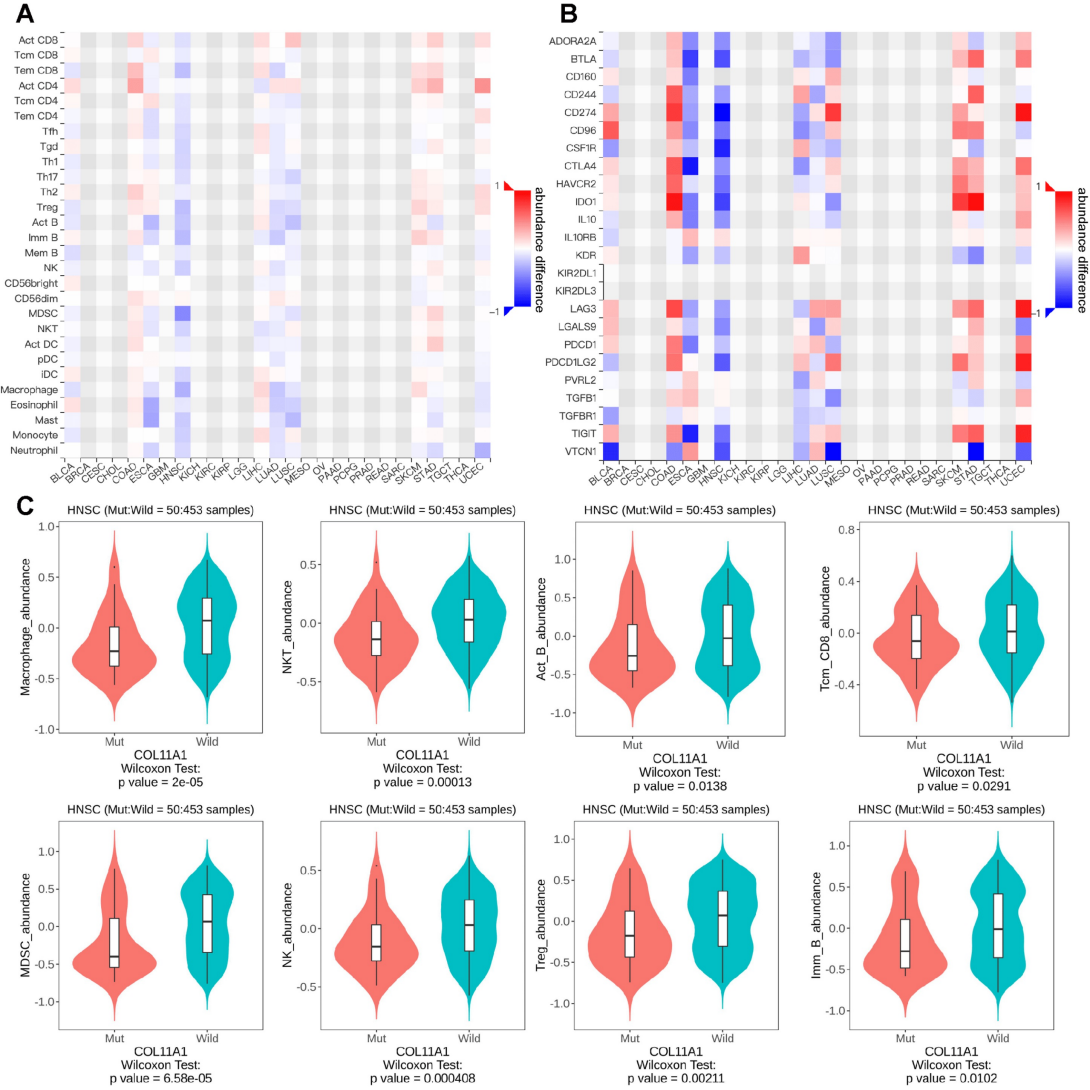
Association of COL11A1 mutation with tumor immune infiltration and chemokine expression TCGA: The Cancer Genome Atlas; HNSC: head and neck squamous carcinoma (A) Utilizing samples from the TCGA-HNSC database, it was found that mutations in COL11A1 were significantly associated with the degree of infiltration by various immune cells.  (B) Based on the samples from the TCGA-HNSC database, the mutation of the COL11A1 gene was found to be significantly associated with the expression of various immune inhibitors and the state of immune cell infiltration. (C) The box plot provides a detailed view, indicating that the infiltration degree of macrophages, NKT cells, etc., was significantly lower in the COL11A1 mutant group compared to the nonmutant group

Considering the significant impact of COL11A1 mutations on immune pathways, we compared the effects of COL11A1 mutations on tumor immune infiltration across various tumor types. Heat maps illustrate the disparities in the degree of infiltration of the majority of immune cells (Figure 5A) and the expression levels of immune checkpoint inhibitors (Figure 5B) in head and neck tumors. Specifically, samples with the COL11A1 mutation exhibited significantly reduced levels of infiltration of key immune cells, including macrophages, NKT cells, active B cells, and natural killer cells, compared to their wild-type counterparts (Figure 5C).

**Figure 6:**
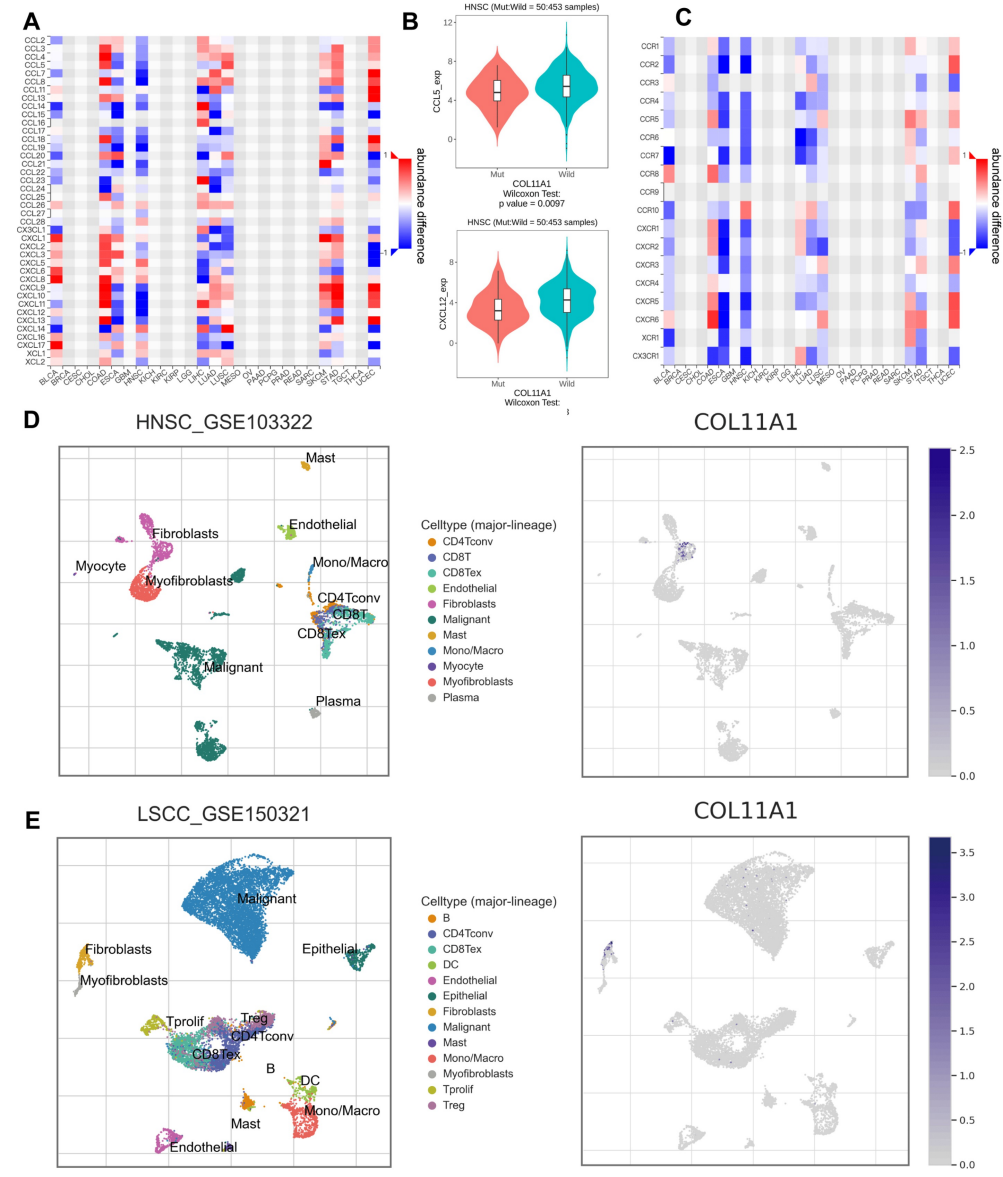
The mutation of COL11A1 is closely related to tumor immunity HNSC: head and neck squamous carcinoma; TCGA: The Cancer Genome Atlas (A) Based on the samples from the TCGA-HNSC database, a significant association was observed between the COL11A1 mutation and the expression levels of multiple chemokines. (B) The box plot illustrates that the expression levels of CCL5 and CCL12 were significantly reduced in the COL11A1 mutant group compared to the nonmutant group. (C) Based on the samples from the TCGA-HNSC database, the mutation of the COL11A1 gene was found to be significantly associated with the expression levels of various chemokine receptors. (D-E) Single-cell sequencing data from GSE103322 (Figure D) and GSE150321 (Figure E) showed that COL11A1 was predominantly expressed in fibroblast cell types, with minimal expression in other cell types

Additionally, given the potential interference of this mutation with chemokine-related signaling pathways, we further investigated the correlation between the COL11A1 mutation status and the expression of chemokines and their receptors in multiple cancers. The findings revealed notable differences in the expression of chemokines and chemokine receptors between the mutant and nonmutant groups(Figure 6A). For instance, significant differences were observed in the expression of CCL5 and CXCL12 between COL11A1-mutated and nonmutated samples (Figure 6B), with similar disparities noted in chemokine receptor genes (Figure 6C). To further investigate the intrinsic relationship between the COL11A1 mutation and chemokine expression differences, we validated the expression of COL11A1 in various cell types using single-cell sequencing datasets GSE103322 (Figure 6D) and GSE150321 (Figure 6E). The results indicated that COL11A1 was predominantly expressed in fibroblasts. Given that fibroblasts, as a primary source of cytokines and chemokines, play a pivotal role in regulating tumor immune responses, this discovery offered a new perspective for exploring the mechanism of their involvement in immune regulation.

COL11A1 mutation's impact on immunity with the estimate and ssGSEA methods

**Figure 7:**
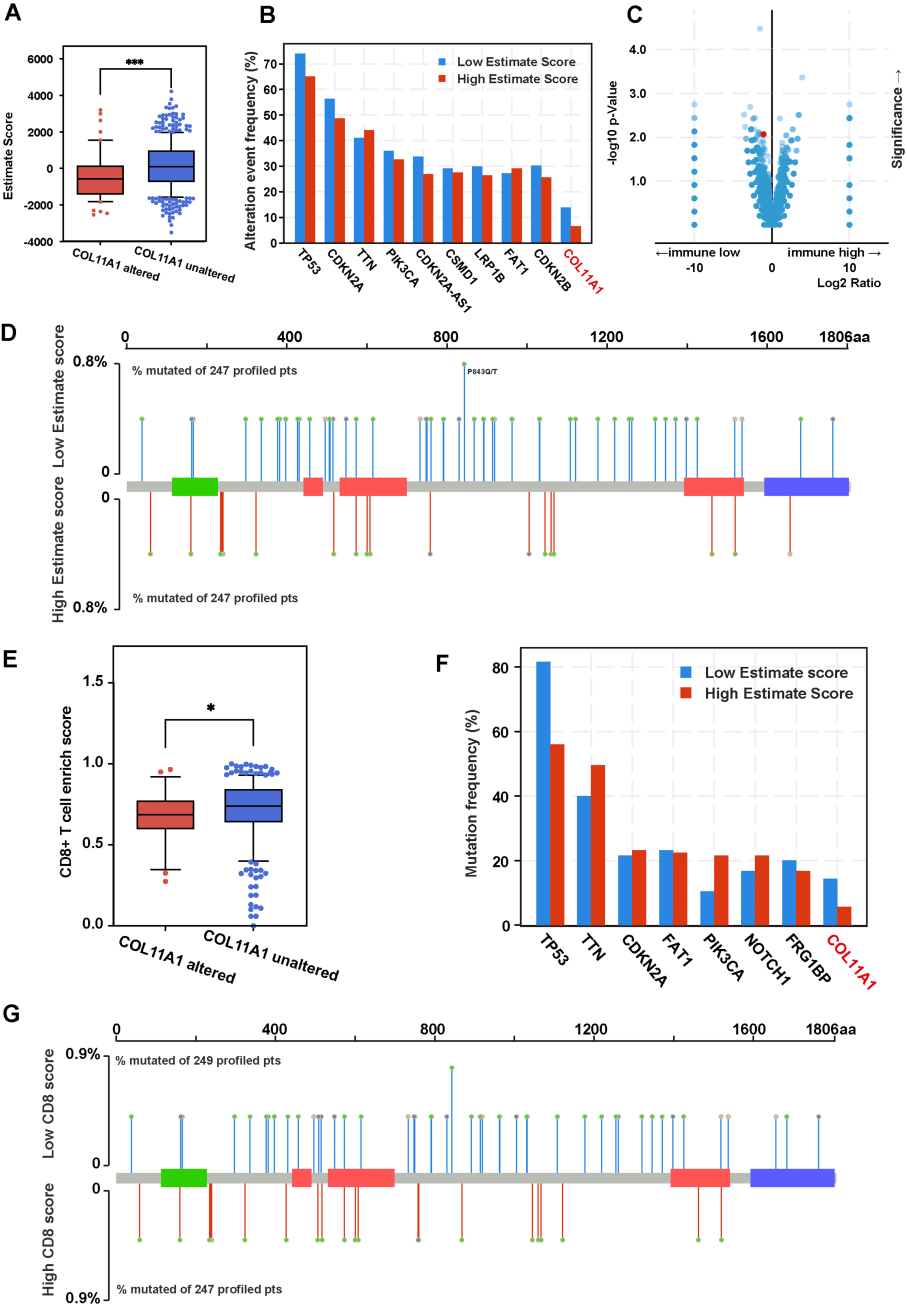
Verification of the relationship between COL11A1 mutation and immunity using Estimate and ssGSEA methods HNSC: head and neck squamous carcinoma; TCGA: The Cancer Genome Atlas; ssGSEA: single sample gene set enrichment analysis (A) The box plot shows that the estimated score of COL11A1 mutant samples is significantly lower than that of the nonmutant samples. (B) The bar graph illustrates the mutation frequency difference of hotspot mutant genes between patients with high and low estimated scores. (C) The volcano plot illustrates that the difference in gene mutation frequency between the high and low immune infiltration groups, with the mutation of COL11A1, was significantly different between the two groups. (D) The mutation frequency and site difference between high and low estimated score samples: the overlap of mutation sites is low, but the mutation frequency difference is considerable. (E) The box plot shows that the CD8 T cell enrichment score of COL11A1 mutant samples is significantly lower than that of nonmutant samples. (F) The bar graph illustrates the mutation frequency difference of hotspot mutant genes between patients with high CD8 T cell enrichment scores and low CD8 T cell enrichment scores. (G) The mutation frequency and site difference between high CD8 T cell enrichment score samples and low CD8 T cell enrichment score samples: the overlap of mutation sites is low, but the mutation frequency difference is considerable. The data used in this section are all based on TCGA-HNSC data

To further elucidate the relationship between COL11A1 mutation and immune infiltration, we employed the EStimate Score, calculated by the EStimate algorithm, as a quantitative index to gauge the extent of immune cell infiltration. Upon comparing the EStimate Score of the COL11A1 mutant group with that of the wild-type group, we found that the score for the COL11A1 mutant group was significantly lower than that of the nonmutant group, suggesting an association with a decreased level of immune cell infiltration (Figure 7A). Moreover, based on the median EStimate Score, we segregated the patient population into a high-score group and a low-score group and evaluated the differences in the gene mutation spectrum between the two groups. The analysis revealed that several genes, including TP53, CDKN2A, TTN, and COL11A1, exhibited a higher mutation frequency trend in the group with lower levels of immune infiltration, particularly COL11A1, with a mutation frequency of 13.79% in the low-score group and 6.51% in the high-score group (Figure 7B). Through statistical testing of the difference in gene mutation frequency between the two groups, we determined that the mutation frequency of COL11A1 exhibited a significant change (Figure 7C). In a detailed analysis of mutation sites in patients in both groups, we observed notable differences in overall mutation frequency between the two groups, even though most specific mutation sites were not identical (Figure 7D).

**Figure 8:**
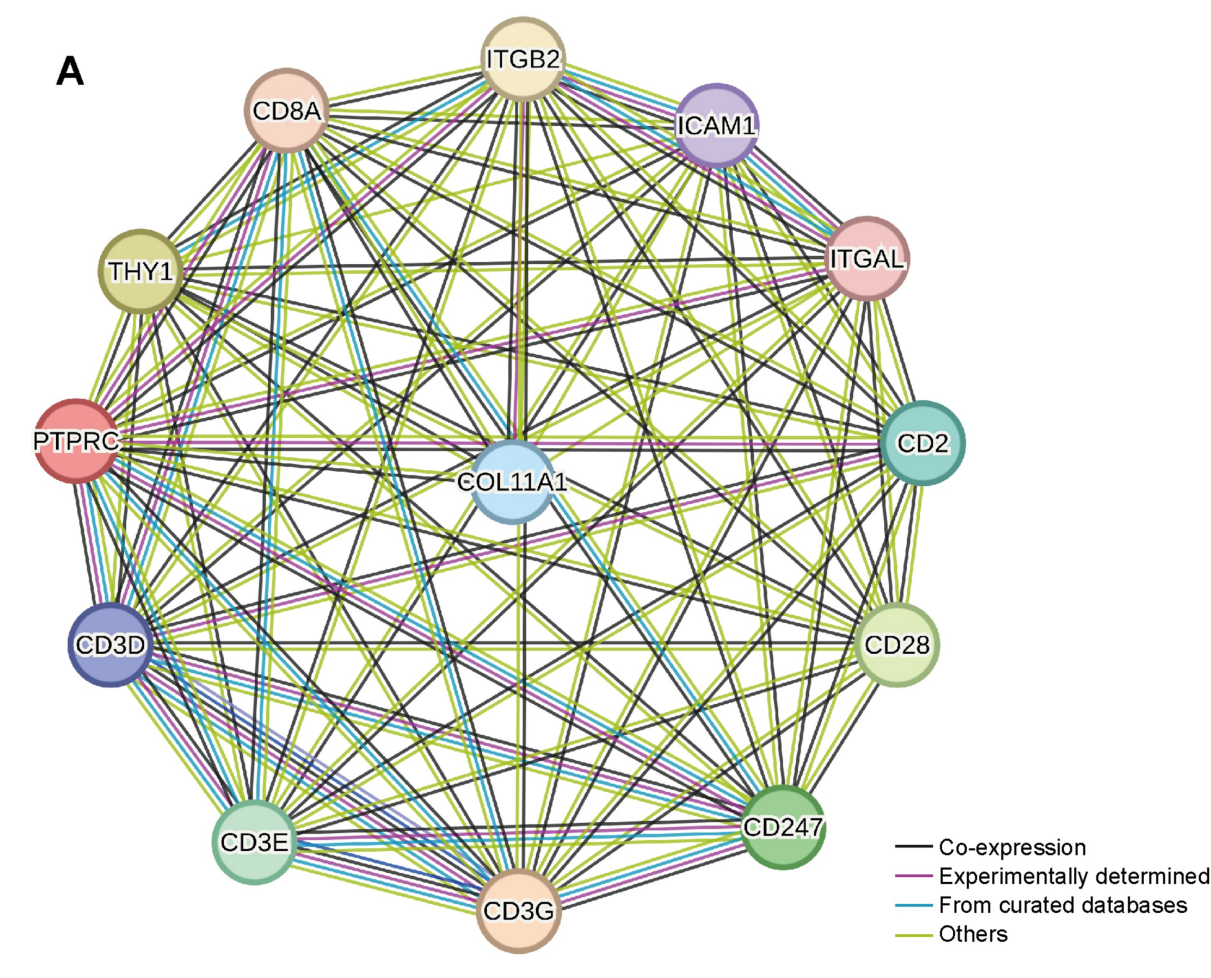
Interaction network of key genes in the CD8+ T cell gene set and COL11A1 (A) The interaction network of crucial genes in the Geneset of CD8+ T cells and COL11A1, where each node represents a gene, and potential interactions are indicated by straight lines

Furthermore, we utilized the ssGSEA algorithm to assess the enrichment of CD8+ T cells in various samples, categorizing the samples into a CD8+ T cell high-score group and a low-score group. The analysis results indicated that the CD8+ T cell scores in the COL11A1 mutant group were lower than those in the nonmutant or low-score group (Figure 7E). By investigating the differences and similarities of gene mutations between the two groups, we discovered that the mutation frequencies of genes such as TP53, TTN, and CDKN2A varied significantly among the different score groups (Figure 7F). A comparison of mutation sites of these significantly different genes revealed that, despite the difference in mutation frequency, the primary mutation sites of the two groups did not coincide (Figure 7G). Additionally, we examined the network of protein interactions between crucial genes in this GeneSet and discovered that COL11A1 exhibits interaction relationships with multiple vital genes (Figure 8A).

## Discussion

In this investigation, the focus was on the role of the TMB and COL11A1 gene in HNC and their correlation with tumor progression. The observed correlation between TMB and tumor staging aligns with prior studies [26], which have consistently shown that lower TMB is associated with early-stage cancers, suggesting more stable genomes. Additionally, the significant relationship between TMB and HPV status, where HPV-positive tumors exhibited higher TMB, corroborates earlier findings [27] that suggest HPV infection leads to a distinct mutational profile, possibly due to viral oncogenic mechanisms. 

The identification of COL11A1 as a commonly mutated gene in HNSCC, despite its relatively lower frequency compared to other genes like TP53 and TTN [28], opens new avenues for understanding its role in tumor progression. Previous studies have largely focused on high-frequency mutations, but our findings indicate that COL11A1 mutations, though less frequent, may have significant prognostic implications. The association of COL11A1 mutations with poorer survival and higher TMB further supports its potential as a marker for HNSC.

Moreover, enrichment analysis of COL11A1 mutation-associated genes emphasized immune-associated pathways, which share similarities with previous work on TP53 and HRAS mutations [29] affecting immune-associated pathways and immune cell infiltration. 

Additionally, significant changes were observed in the expression of chemokines and chemokine receptors in tumor tissues with COL11A1 mutations, suggesting that COL11A1 mutations might affect chemotaxis signaling in the tumor microenvironment, thereby influencing immune cell invasion. We also used various methods to verify the relationship between COL11A1 mutation and immune infiltration, further validating our observations. These findings provide new insights into the role of COL11A1 in HNC and its potential role in tumor immune evasion. 

Finally, while our study provides valuable insights into the role of COL11A1 in HNSCC, it is essential to consider the broader implications across different types of cancers and diseases. COL11A1 has been implicated in various conditions beyond cancer, including fibrotic disorders, where it may exert similar or distinct effects. Exploring these parallels can offer a more comprehensive understanding of COL11A1's multifaceted roles and potentially identify common therapeutic targets.

Although this study reveals the important role of COL11A1 mutations in HNC, there are still some limitations. First, this study relies on data from public databases, which may have sample bias or data quality issues. Second, although we observed an association between COL11A1 mutations and TMB and prognosis, the causal relationship was not clear. In addition, due to the lack of functional experimental verification, the specific effects of COL11A1 mutation on the tumor immune microenvironment still need to be further explored.

Our future studies should focus on the following aspects: First, further verify the causal relationship between COL11A1 mutation and TMB and immune escape, especially to reveal its molecular mechanism through functional experiments [30]. Secondly, explore the potential of COL11A1 mutation as a biomarker for immunotherapy and evaluate its application value in clinical practice. Finally, expand the sample size of the study, especially to include more patients of different races and regions, to enhance the universality of the results and reveal possible racial or environmental differences.

In summary, this study underscores the significant role of TMB and COL11A1 in HNC, offering novel insights for further understanding the pathogenesis of HNC and the development of new therapeutic strategies.

## Conclusions

Our analysis showed that significant differences were observed in TMB across various clinical characteristics of patients with HNC, with higher TMB being associated with poorer survival outcomes. Furthermore, the COL11A1 gene exhibits a high mutation frequency in HNCs, and its mutations may significantly impact patient survival while correlating with elevated TMB. Immuno-infiltration analysis also revealed that COL11A1 mutations might alter the tumor microenvironment by influencing immune cell infiltration.
